# First-Line Anaplastic Lymphoma Kinase (ALK) Inhibitors for ALK-Positive Lung Cancer in Asian Populations: Systematic Review and Network Meta-Analysis

**DOI:** 10.3390/jcm10194376

**Published:** 2021-09-25

**Authors:** Kuan-Li Wu, Hsiao-Ling Chen, Ying-Ming Tsai, Tai-Huang Lee, Hsiu-Mei Chang, Yu-Chen Tsai, Cheng-Hao Chuang, Yong-Chieh Chang, Yu-Kang Tu, Chih-Jen Yang, Jen-Yu Hung, Inn-Wen Chong

**Affiliations:** 1 Division of Pulmonary and Critical Care Medicine, Department of Internal Medicine, Kaohsiung Medical University Hospital, Kaohsiung Medical University, Kaohsiung 80708, Taiwan; 1070476@kmuh.org.tw (K.-L.W.); yingming@kmu.edu.tw (Y.-M.T.); weatlee@gmail.com (T.-H.L.); 1010362@kmuh.org.tw (Y.-C.T.); 1040239@kmuh.org.tw (C.-H.C.); jyhung@kmu.edu.tw (J.-Y.H.); 2 Department of Pharmacy, Kaohsiung Municipal Ta-Tung Hospital, Kaohsiung Medical University, Kaohsiung 80145, Taiwan; 1058065@kmuh.org.tw (H.-L.C.); 880504@kmhk.org.tw (H.-M.C.); 980770@kmuh.org.tw (Y.-C.C.); 3 Department of Internal Medicine, School of Medicine, College of Medicine, Kaohsiung Medical University, Kaohsiung 80708, Taiwan; 4 Department of Internal Medicine, Kaohsiung Municipal Ta-Tung Hospital, Kaohsiung Medical University, Kaohsiung 80145, Taiwan; 5 Institute of Epidemiology and Preventive Medicine, National Taiwan University, Taipei 100025, Taiwan; yukangtu@ntu.edu.tw; 6 Department of Medical Research, National Taiwan University Hospital, Taipei 100025, Taiwan; 7 Faculty of Post-Baccalaureate Medicine, College of Medicine, Kaohsiung Medical University, Kaohsiung 80708, Taiwan; 8 Department of Respiratory Therapy, College of Medicine, Kaohsiung Medical University, Kaohsiung 80708, Taiwan

**Keywords:** Asian, anaplastic lymphoma kinase inhibitor, non-small cell lung cancer

## Abstract

Various anaplastic lymphoma kinase inhibitors (ALKIs) have been approved for first-line use in treating anaplastic lymphoma kinase (ALK)-rearranged non-small cell lung cancer (NSCLC). To date, no head-to-head comparison of these newer generation ALKIs has been made, and different efficacies of ALKIs may present across ethnicity. This study aims to compare newer generation ALKIs for treatment efficacy in Asian groups using network meta-analysis. Phase II/III trials that enrolled treatment-naïve Asian ALK-rearranged NSCLC patients treated by ALKIs were included. Progression-free survival (PFS) and overall response rate (ORR) of each trial were extracted as indicators of drug efficacy. Surfaces under cumulative ranking curves (SUCRAs) were calculated as a numeric presentation of the overall ranking associated with each agent. After a systematic literature review, six phase III clinical trials were included. Our results showed that newer generation ALKIs, such as alectinib, brigatinib, ensartinib, and lorlatinib, all demonstrated superior efficacy to crizotinib. Among those, ensartinib exhibited the best overall SUCRA value and ranked first among all agents. According to our network meta-analysis, ensartinib may currently be the most effective first-line treatment for Asian patients with ALK-positive NSCLC. However, this conclusion needs further validation by a larger scale of clinical trials or posthoc analysis of Asian populations. Moreover, in our comparison, low-dose alectinib (300 mg twice daily) exhibited an efficacy profile similar to a higher dose regimen in Asian populations.

## 1. Introduction

In 2020, lung cancer accounted for 1.8 million deaths globally, ranking first among all causes of cancer death [1]. Upon diagnosis, 50% of patients have distant metastases, with a 5-year survival rate lower than 5% [2]. No cure has been achieved for later-stage patients, making lung cancer the most devastating cancer category.

Among all forms of lung cancer, 85% of cases are histologically sorted as non-small cell lung cancer (NSCLC) and 15% as small cell lung cancer (SCLC). NSCLC is further subdivided into three major entities: adenocarcinoma, squamous cell carcinoma, and large cell carcinoma [3]. Beyond histologic differences, interpatient heterogeneity for genetic alteration is well recognized. Some of these so-called targetable driver mutations have been linked to the susceptibility of molecular-targeted therapy, creating a new era for lung cancer treatment [4]. Since the early 2000s, the development of epidermal growth factor receptor (EGFR) tyrosine kinase inhibitors (TKIs) has changed our management of patients harboring sensitizing driver mutations due to the impressive efficacy and better tolerability of these drugs. Subsequently, an increasing number of driver oncogenes were identified, and their corresponding targeting agents were synthesized. To date, seven targetable genetic alterations in lung cancer have been listed in the latest National Comprehensive Cancer Network (NCCN) guidelines. However, such genetic alterations exist exclusively among NSCLC patients [5].

Anaplastic lymphoma kinase (ALK) rearrangement, mostly echinoderm microtubule-associated protein-like 4 (EML4)-ALK fusions, is the second most common targetable genetic alteration in NSCLC, with an estimated prevalence of 3% to 5% [6]. As a human receptor tyrosine kinase, ALK activates downstream signals involving cell survival, proliferation, and apoptosis evasion [7]. First-generation ALKI crizotinib has been approved by the United States Food and Drug Administration (FDA) since 2011 [8]. Later, crizotinib became the first standard ALKI for untreated ALK-rearranged NSCLC patients based on its superior efficacy, as evidenced by data from the PROFILE 1014 study. Afterward, several second-generation ALKIs—ceritinib, alectinib, and brigatinib—along with a third-generation inhibitor, lorlatinib, were developed and approved for clinical use. Newer generation ALKIs displayed a better survival benefit and safety profile than the first-generation ALKI crizotinib [9]. However, no head-to-head studies comparing newer generation ALKIs with each other had been conducted.

Ethnic differences among lung cancer patients present a critical issue in many aspects, including genetic characteristics, treatment response, drug toxicity, and prognosis [10,11]. For example, the FLAURA study found a discrepancy in treatment efficacy between Asian and non-Asian populations; the study demonstrated a better hazard ratio (HR) for progression-free survival (PFS) using osimertinib for Caucasians with EGFR-mutant NSCLC patients in the first-line setting; the same efficacy was not observed in Asian populations [12]. Recently, for ALK-rearranged NSCLC patients, the CROWN trial demonstrated that lorlatinib attributes to a relatively lower hazard ratio of disease progression in non-Asian subjects (HR:0.19, CI: 0.11–0.32) than in Asian subjects (HR:0.47, CI: 0.27–0.82) [13]. Therefore, results from large-scale clinical trials employing Caucasians as the majority of subjects may not be fully applicable to Asian populations. A comprehensive study on the use of ALKIs specifically in Asian populations would be valuable. Two phase III clinical trials, J-ALEX and ALESIA, enrolled only Asian subjects to evaluate the efficacy of alectinib [14,15]. However, for other ALKIs, no trials enrolling exclusively Asian participants have been conducted. The best first-line ALKI for Asian NSCLC patients remains to be determined.

Therefore, the present study aims to provide the comparative efficacies of different ALKIs in untreated Asian ALK-rearranged NSCLC patients, using network meta-analysis to retrieve efficacy profiles from trials that enrolled Asian subjects partially or exclusively.

## 2. Materials and Methods

This meta-analysis was performed in accordance with the PRISMA (Preferred Reporting Items for Systematic Reviews and Meta-Analyses) guidelines [16]. The protocol for systematic review was created and registered on the PROSPERO website (Registration No. CRD42021228647) available online: https://www.crd.york.ac.uk/prospero/display_record.php?RecordID=228647; accessed on 7 March 2021.

### 2.1. Search Strategy

PubMed, Embase, Cochrane Reviews, and Clinical-Trials.gov were searched up to 20 September 2021 without language limitations to identify eligible studies. In addition, we searched abstracts from global and Asian oncology congresses for updated study reports. This strategy is presented in Appendix A. The search terms used in the search strategy included non-small cell lung cancer (NSCLC), anaplastic lymphoma kinase (ALK) inhibitors, and tyrosine kinase inhibitors (crizotinib, ceritinib, alectinib, brigatinib, ensartinib, and lorlatinib). The references of related reviews were also checked to include more relevant studies.

### 2.2. Study Selection

Two independent reviewers (H.L. Chen and K.L. Wu) selected the articles. In the case of any disagreements, a third reviewer (C.J. Yang) was included in the discussion to reach a consensus. Study eligibility was defined according to the following criteria: (1) completed phase II–III randomized clinical trials (RCTs); (2) patients with ALK-positive advanced or metastatic NSCLC; (2) patients who were never treated with ALKIs prior to recruitment; (3) RCTs providing efficacy and safety information on various ALKIs; (4) RCTs focusing on Asian populations or providing subgroup analysis for Asian groups.

### 2.3. Data Extraction and Quality Assessment

The extracted information included trial name, year, trial phase, previous treatment, baseline characters, intervention, and sample size. Efficacy was presented by progression-free survival (PFS) and objective response rate (ORR). The risk of bias (ROB) assessment tool from the Cochrane Collaboration was used for quality assessment.

### 2.4. Data Synthesis and Statistical Analysis

The hazard ratio (HR) was regarded as a measure of effect size for PFS, and the response ratio (RR) was used as an indicator for ORR.

#### 2.4.1. Pairwise Meta-Analysis

Pairwise meta-analysis was performed for comparisons between first-generation (crizotinib) and new-generation ALKIs. The DerSimonian and Laird random-effects model was used, assuming that a common intervention effect is relaxed and that the effect sizes have a normal distribution. The hazard ratio (HR) was regarded as a measure of effect size for PFS, and the response ratio was used as an indicator for ORR. *I*^2^ statistics were used to measure the proportion of total variation in study estimates due to heterogeneity rather than chance. Pairwise meta-analysis was performed by Review Manager Version 5.4, and subgroup analysis was performed by types of ALKIs.

#### 2.4.2. Network Meta-Analysis

Firstly, network graphs were generated for PFS and ORR separately to identify which treatments were compared directly or indirectly. We then conducted a network meta-analysis (NMA) utilizing the frequentist approach under the fixed-effect model. Contrast-based analysis was performed for multiple treatment comparisons under the restricted maximum likelihood approach. Finally, surfaces under cumulative ranking curves (SUCRAs) were calculated. SUCRA is a numeric presentation of the overall ranking and presents a single number associated with each treatment; SUCRA values range from 0 to 100%. A larger SUCRA value represents a higher rank based on intervention effects. Network meta-analysis was performed using the multivariate random-effects analysis (mvmeta) Stata command (version 16, Stata, College Station, TX, USA).

## 3. Results

### 3.1. Literature Search

**Figure 1:**
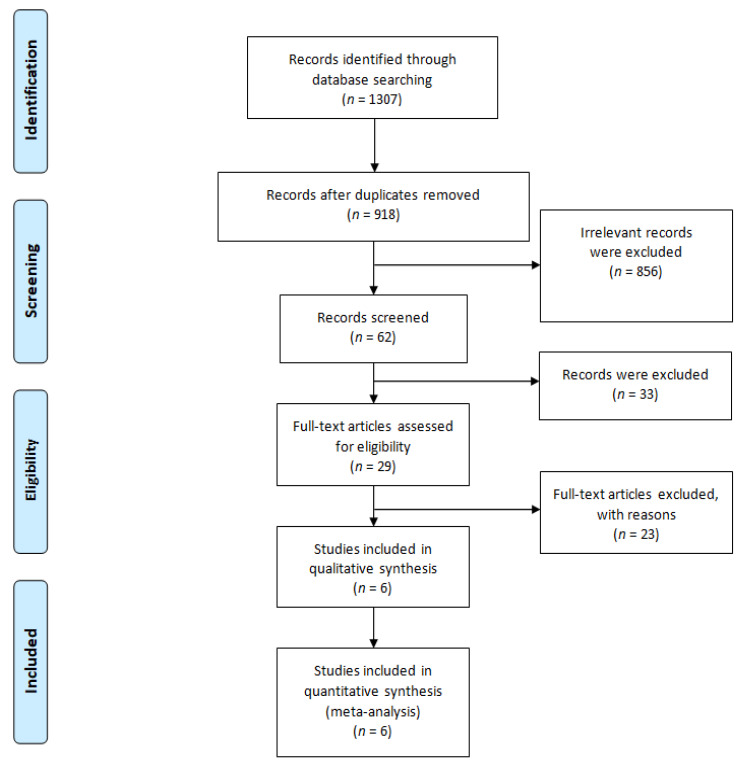
The PRISMA flow diagram.

After searching the listed databases, we found a total of 1307 studies using the search keywords. Among them, 389 studies were removed due to duplicates, 856 studies were removed by title/abstract screening. 33 studies were removed due to unavailable full text and 23 non-relevant articles were removed by full text reading. Finally,. six studies met our inclusion criteria and were selected for qualitative synthesis and quantitative meta-analysis. The PRISMA flow diagram is presented in Figure 1.

### 3.2. Study Characteristics and Quality Evaluation

The study characteristics of the six included phase III RCTs are presented in Table 1. All of the studies were conducted with patients with ALK-rearranged NSCLC without exposure to prior ALKIs. Head-to-head comparisons were performed between crizotinib and new-generation ALKIs. The ALTA-1L trial investigated brigatinib, the CROWN trial investigated lorlatinib, the eXalt3 trial studied ensartinib, and the remaining three RCTs investigated alectinib with a high (600 mg twice daily in the ALESIA and ALEX trials) or low (300 mg twice daily in the J-ALEX trial) dose. The median age was 49–60 years, and the percentage of male patients across the trials ranged from 37% to 55%; 14% to 42% of the included subjects had brain metastasis at baseline. The results of the quality assessment are presented in Appendix B. All of the included trials carried a high risk of performance bias due to their open-label designs. However, the risk of detection bias was lower because a blinded independent review committee had assessed disease progression or treatment response in each trial. Quality was unclear with regards to sequence generation, allocation concealment, and selective reporting due to the lack of detailed information.

### 3.3. Pooled Results for Generation Differences in Efficacy

The pooled results of generation differences in efficacy and safety are provided in Appendix C. Compared to the first generation ALKI (crizotinib), new-generation ALKIs significantly increased PFS (HR = 0.38, 95% confidence interval (CI) = 0.32–0.47 and ORR = 1.14, 95% CI = 1.05–1.22).

### 3.4. Efficacy Evaluation from the Network Meta-Analysis

**Figure 2:**
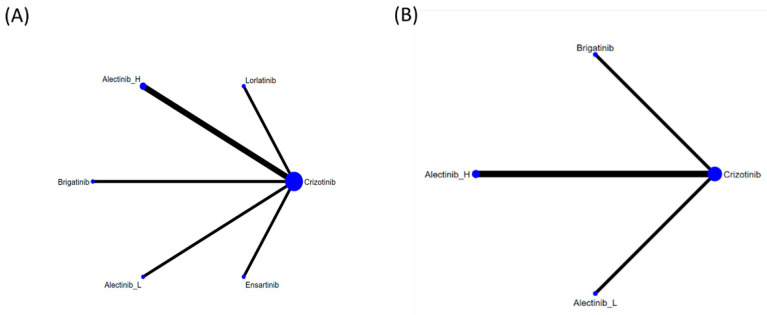
Network constructions for comparisons in PFS and ORR: (**A**) network constructions for PFS, (**B**) network constructions for ORR.

**Figure 3:**
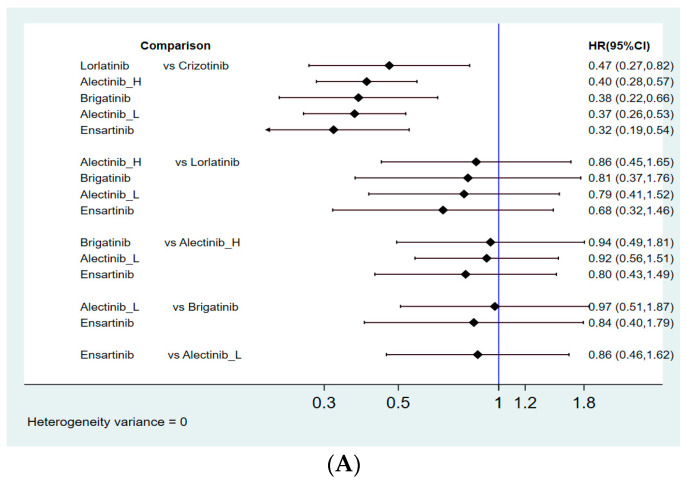

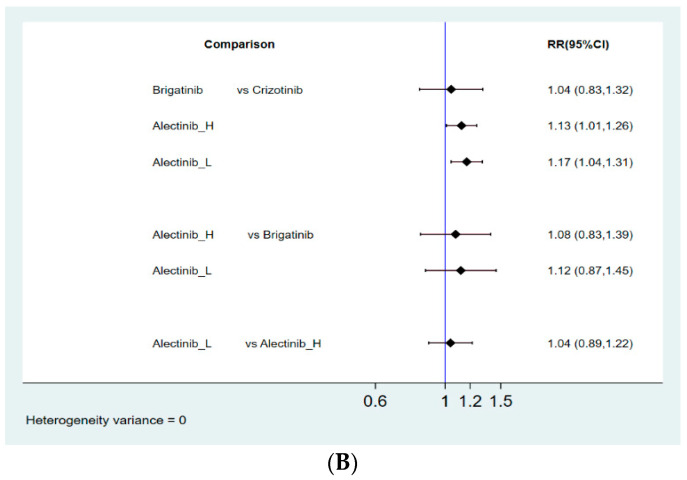
Summary of effect sizes for efficacy comparison: (**A**) pairwise comparisons for PFS; (**B**) pairwise comparisons for ORR.

Figure 2 presents the network constructions of eligible comparisons. To understand the dose-effect of alectinib, high (600 mg twice daily) and low (300 mg twice daily) doses were regarded as different interventions. For PFS, six interventions were included in the network constructions: crizotinib, brigatinib, ensartinib, lorlatinib, and high- and low-dose alectinib. Due to a lack of information on Asian patients, ensartinib and lorlatinib were excluded from the network constructions for ORR analysis. The effect sizes of pairwise comparisons are summarized in Figure 3 and SUCRA rankings in Appendix D and the probability of being the best treatment (based on all efficacy indicators) in Appendix E.

#### 3.4.1. Progression-Free Survival

All 4 new-generation ALKIs (alectinib, brigatinib, ensartinib, and lorlatinib) analyzed in our study had superior effects compared with crizotinib (HR = 0.40, 95% CI = 0.28–0.57 for high-dose alectinib; HR = 0.37, 95% CI = 0.26–0.53 for low-dose alectinib; HR = 0.38, 95% CI = 0.22–0.66 for brigatinib; HR = 0.47, 95% CI = 0.27–0.82 for lorlatinib; HR = 0.32, 95% CI = 0.19–0.54 for ensartinib). Among alectinib, brigatinib, lorlatinib, and ensartinib, no superiority could be detected in one drug compared to the other. In addition, low-dose alectinib (300 mg twice daily) showed efficacy similar to high-dose alectinib (600 mg twice daily) with respect to PFS (HR = 0.92, 95% CI = 0.56–1.51) in the paired comparison. Moreover, ensartinib demonstrated the best PFS with the highest SUCRA values (SUCRA = 79.1%), followed by low-dose alectinib (SUCRA = 65.3%), brigatinib (SUCRA = 61.1%), high-dose alectinib (SUCRA = 54.7%), and lorlatinib (SUCRA = 39.0%).

#### 3.4.2. Objective Response Rate

Compared to crizotinib, alectinib showed better ORRs (RR = 1.13, 95% CI = 1.01–1.26 for high-dose alectinib; RR = 1.17, 95% CI = 1.04–1.31 for low-dose alectinib) but not brigatinib (RR = 1.04, 95% CI = 0.83–1.32). However, no significantly superior effects were found in high- or low-dose alectinib versus brigatinib (RR = 1.08, 95% CI = 0.83–1.39 for high-dose alectinib; RR = 1.12, 95% CI = 0.87–1.45 for low-dose alectinib) in a paired comparison. Moreover, low-dose alectinib demonstrated a similar effect on ORRs as high-dose alectinib (RR:1.04, 95% CI = 0.89–1.22). Ranked by SUCRA and Prbest value for ORR, low-dose alectinib was rated best (SUCRA = 82.6%, Prbest = 57.8%), followed by high-dose alectinib (SUCRA = 67.7%,), brigatinib (SUCRA = 37.6%,), and crizotinib (SUCRA = 12.1%,).

## 4. Discussion

The present meta-analysis extracted limited existing data from trials involving Asian subjects for testing the efficacy of ALKIs and demonstrated that ensartinib provides the best PFS, while alectinib shows the best ORR.

Earlier-developed ALKIs, such as crizotinib and ceritinib, have demonstrated antitumor activity superior to conventional chemotherapy in the global trials PROFILE 1014 and ASCEND-4 [22,23]. In the Asian subgroup, the HRs for median PFS were 0.40–0.44 for subjects using crizotinib [22,24] and 0.66 for subjects using ceritinib [23]. Since the results were robust, no trials have focused on the efficacy of newer ALKIs, such as alectinib, brigatinib lorlatinib, and ensartinib, compared with chemotherapy. Moreover, ALKIs have been established as the first-line treatment option for ALK-rearranged NSCLC patients. Hence, in the present network meta-analysis, trials comparing older ALKIs with chemotherapy as the comparator were excluded.

Despite its tremendous therapeutic efficacy, drug resistance to crizotinib develops eventually in almost all cases. Most often, secondary ALK gene mutations in the kinase domain, such as L1196M, G1269A, and G1202R, account for the mechanism of drug resistance [25]. Furthermore, the occurrence of secondary mutations seems to differ by ethnicity. Yanagitani et al. reported a 67% secondary mutation rate in Japanese ALK-positive patients receiving crizotinib, compared with 20–50% in Caucasian patients. The difference may be explained by relatively higher plasma concentrations of crizotinib among Japanese patients [26].

Newer generation ALKIs, such as ceritinib, alectinib, ensartinib, and brigatinib, exhibit a different spectrum of ALK resistance mutations; these newer generation ALKIs have been indicated for NSCLC patients who fail treatment with crizotinib. Moreover, ceritinib, alectinib, brigatinib, and lorlatinib have been recommended by the NCCN guidelines for first-line use due to their superior efficacy in large phase III RCTs.

In the ALEX trial, the use of alectinib 600 mg twice daily demonstrated similar efficacy in Asian and non-Asian groups [27]. In our study, alectinib demonstrated the best ORR and second-best PFS profiles among the newer generation ALKIs for Asian patients. Surprisingly, alectinib 300 mg twice daily exhibited superior SUCRA and Prbest values to the regimen of 600 mg twice daily. Due to the legal limitation on sodium lauryl sulfate levels in Japan, dose escalation was stopped at 300 mg twice daily in the Japanese phase I/II study (AF-001JP) [28], leading to the approved dose of 300 mg twice daily by the Japanese authority [15]. Theoretically, the safety profile should be improved at a reduced dose. However, this hypothesis could not be proven by this study due to limited resource data. Looking into the J-ALEX trial, the rate of adverse events (grade >= 3) was 36.9%, which is higher than the 29% in the ALESIA study [14,15]. In a real-world retrospective study in Japan, the overall rate of grade >= 3 adverse events was only 10.1% among the 1211 subjects using alectinib 300 mg twice daily, regardless of the treatment setting [29]. Therefore, alectinib 300 mg twice daily may present a better option for Asian groups in terms of both efficacy and safety.

Our meta-analysis contains several limitations. First, data associated with the treatment outcome of ALKIs among Asian groups are significantly more abundant for alectinib than for other ALKIs. ALESIA and J-ALEX were trials that only enrolled Asian subjects, and 45% of the patients enrolled in the ALEX study were Asian. However, there were fewer Asian cases enrolled in the ALTA-1L, CROWN, and eXalt3 trials, respectively, which may introduce bias to over-estimate the superiority of ensartinib. Moreover, this imbalance in research may overrepresent the effect of alectinib. Second, overall survival information was lacking in the RCTs that enrolled ALK-positive patients, hampering the extrapolation of our treatment rankings to long-term outcomes. Moreover, information about efficacy on brain metastasis and safety profiles among Asian groups could not be retrieved. Therefore, comparisons relating to brain metastasis control and safety issues were not achievable in this study. Third, the heterogeneity among trials and the nature of our indirect comparison design may over-simplify the results of our study. However, our study’s focus on ALKI-naïve patients of Asian ethnicity should have decreased the heterogeneity.

Ultimately, our study results serve as a platform for further clinical research on new-generation ALKIs as first-line therapies for advanced ALK-positive NSCLC patients of Asian ethnicity and not as direct evidence to establish any single agent as a frontline option at present.

## 5. Conclusions

In the first-line setting, ensartinib may currently present as the most effective ALKI for PFS based on the findings of our network meta-analysis. Alectinib is second to ensartinib in terms of PFS, but it demonstrated the best ORR for Asian ALK-rearranged NSCLC patients. The efficacy profile of alectinib is also supported by the most abundant cases at present. Moreover, low-dose alectinib (300 mg twice daily) exhibits a similar efficacy profile among Asian populations (specifically, in Japanese patients). However, more randomized controlled trials focusing on Asian populations with regard to drug efficacy, safety profile, and control of brain metastasis are needed to establish the best ALKI option for these patients.

## Figures and Tables

**Table 1:** Characteristics of the included studies of first-line ALKI treatment for advanced ALK-positive NSCLC in the Asian group.

Trial	CROWN [13]		ALTA-1L [17,18]		ALEX [19] (Asian Subgroup)		J-ALEX [20]		ALESIA [15]		eXalt3 [21]	
Author	Shaw et al.		Camidge et al.		Camidge et al.		Nakagawa et al.		Zhou et al.		Horn et al.	
Year	2020		2020		2019		2020		2019		2021	
Design	Phase III, Open Label, RCT		Phase III, Open Label, RCT		Phase III, Open Label, RCT		Phase III, Open Label, RCT		Phase III, Open Label, RCT		Phase III, Open Label, RCT	
Intervention	Lorlatinib 100 mg qd	Crizotinib 250 mg bid	Brigatinib 90 mg qd for 7 Days, then 180 mg qd	Crizotinib 250 mg bid	Alectinib 600 mg bid	Crizotinib 250 mg bid	Alectinib 300 mg bid	Crizotinib 250 mg bid	Alectinib 600 mg bid	Crizotinib 250 mg bid	Ensartinib 225 mg qd	Crizotinib 250 mg bid
**Sample size**	66	65	59	49	69	69	103	104	125	62	73	78
**Outcome**												
PFS (month)	NA	NA	NR (NR)	11.1 (9.2–NR)	34.8	9.6	34.1	10.2	NR	10.7	NA	NA
HR PFS	0.47 (0.27–0.82)		0.38 (0.22–0.65)		0.43 (0.27–0.67)		0.37 (0.26–0.52)		0.37 (0.22–0.61)		0.32 (0.19–0.55)	
OS (month)	NA	NA	NA	NA	NA	NA	NR	43.7	NR	NR	NA	NA
HR OS	NA		NA		0.74 (0.40–1.36)		0.80 (0.35–1.82)		0.28 (0.12–0.68)		NA	
ORR (%)	NA	NA	75%	71%	81.20%	76.8%	92%	79%	91%	77%	NA	NA
CNS PFS (month)	NA	NA	NR	9.2	NA	NA	NA	NA	NA	NA	NA	NA
DOR (month)	NA	NA	NA	NA	NA	NA	NR	NR	NR	9.3	NA	NA
**Safety**												
AE ≥ grade 3	NA	NA	NA	NA	NA	NA	36.9%	60.6%	29%	48%	NA	NA
Discontinuation due to AE (%)	NA	NA	8.50%	6.30%	13.00%	11.60%	11.7%	23.1%	7%	10%	NA	NA
**Patient character**												
Age (median)	NA	NA	NA	NA	NA	NA	61	59.5	51	49	NA	NA
Male (%)	NA	NA	NA	NA	NA	NA	40%	39%	51%	55%	NA	NA
ECOG 0~1 (%)	NA	NA	NA	NA	NA	NA	98%	98%	97%	98%	NA	NA
Brain metastasis (%)	NA	NA	NA	NA	NA	NA	14%	28%	35%	37%	NA	NA
Stage IV (%)	NA	NA	NA	NA	NA	NA	74%	72%	90%	94%	NA	NA
Smoking, ever (%)	NA	NA	NA	NA	NA	NA	46%	41%	33%	27%	NA	NA

Abbreviations: AE = adverse event; bid = twice daily; CNS = central nervous system; DOR = duration of response; HR = hazard ratio; NA = not available; NR = not reached; ORR = overall response rate; OS = overall survival; PFS = progression-free survival; qd = daily; RCT = randomized controlled trial.

**Figure A1:**
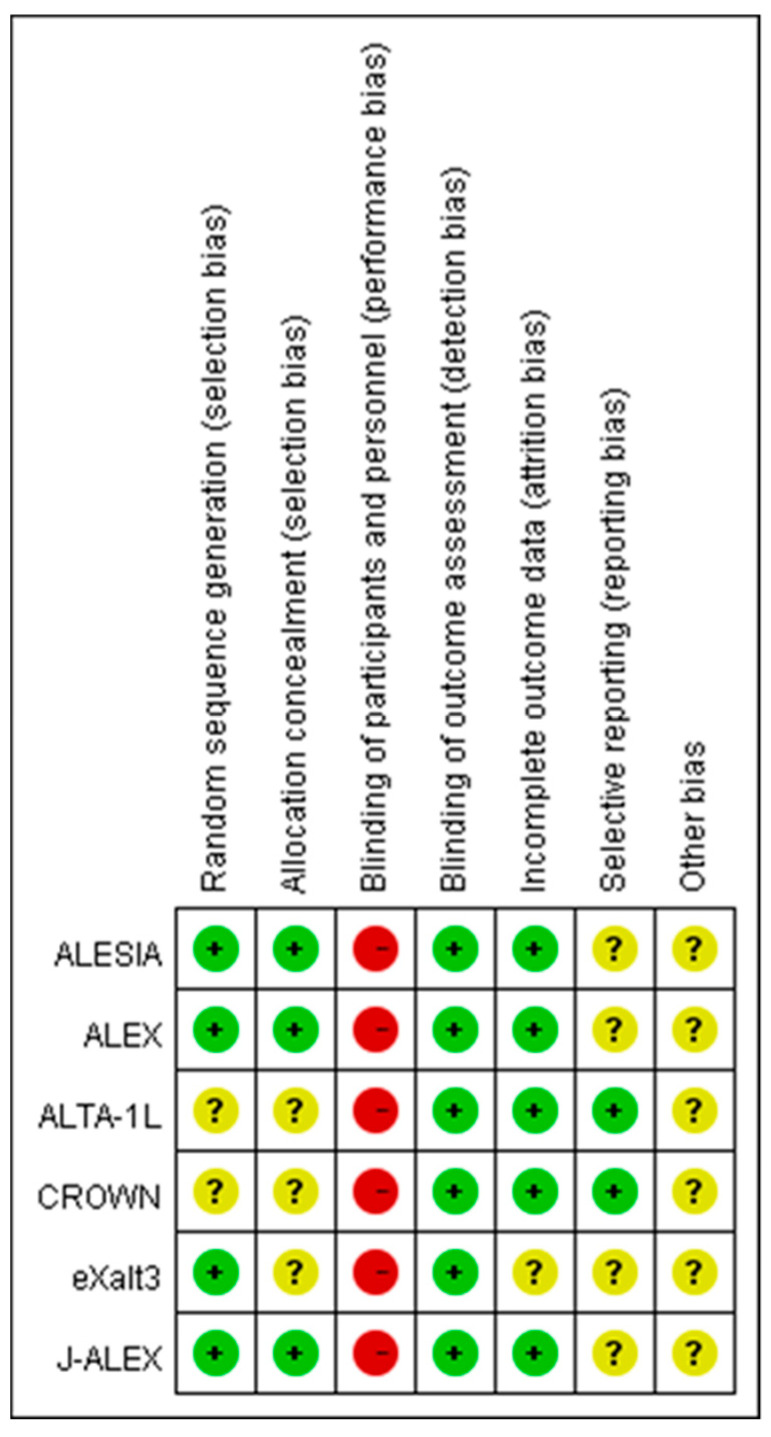

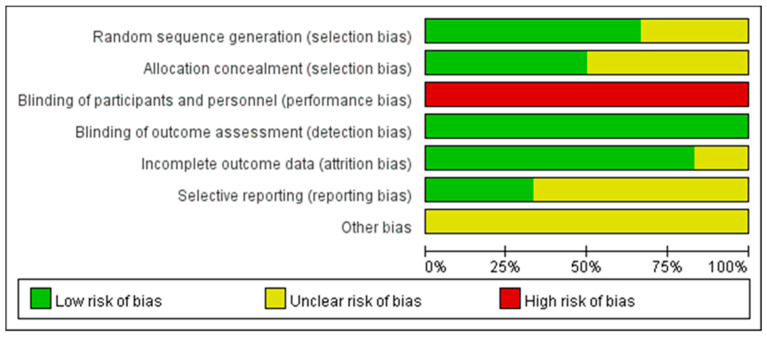
Risk of bias assessment.

**Figure A2:**
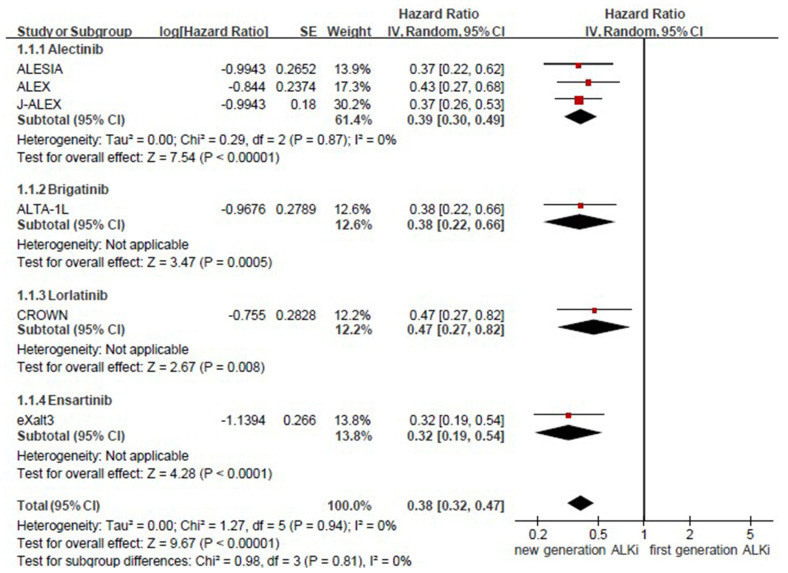
Forest plot and pooled hazard ratio for progression free survival (PFS).

**Figure A3:**
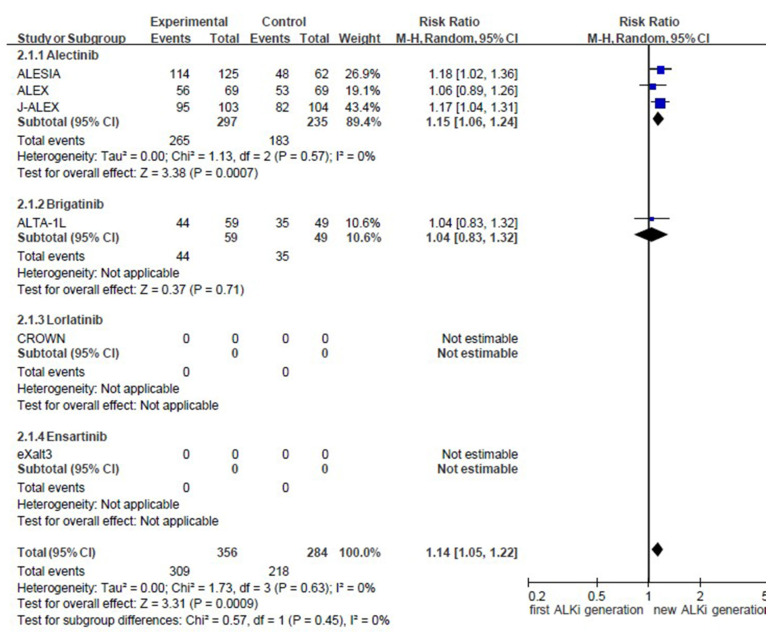
Forest plot and pooled response ratio for objective response rate (ORR).

**Figure A4:**
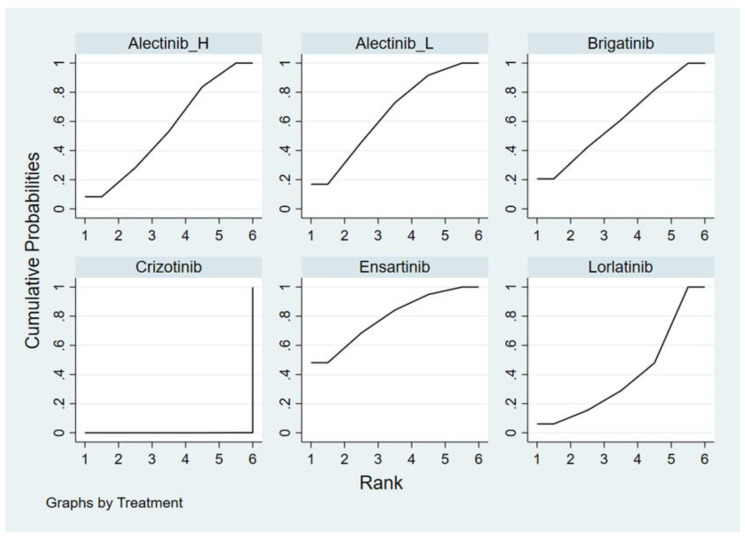
Cumulative ranking probability for progression free survival (PFS).

**Figure A5:**
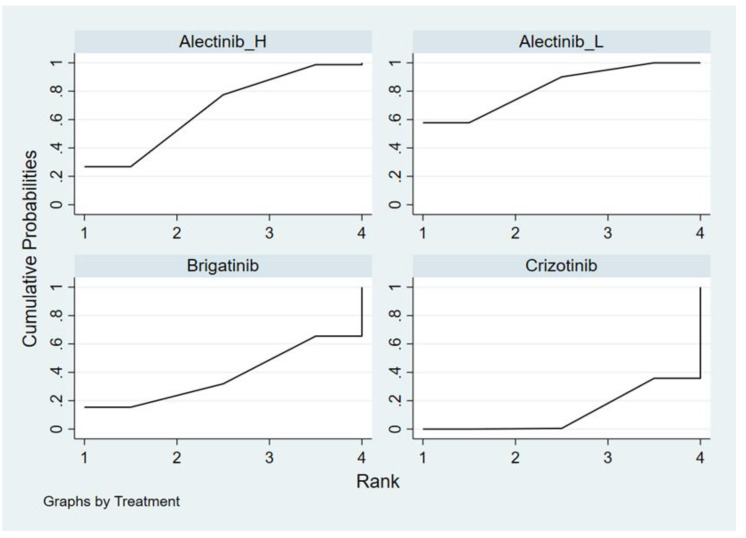
Cumulative ranking probability for objective response rate (ORR).

**Figure A6:**
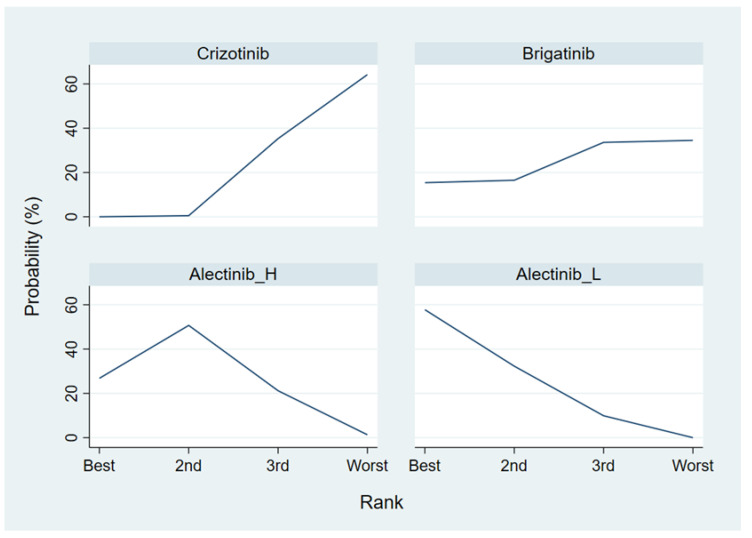
Probability of being the best treatment with regard to progression free survival (PFS).

**Figure A7:**
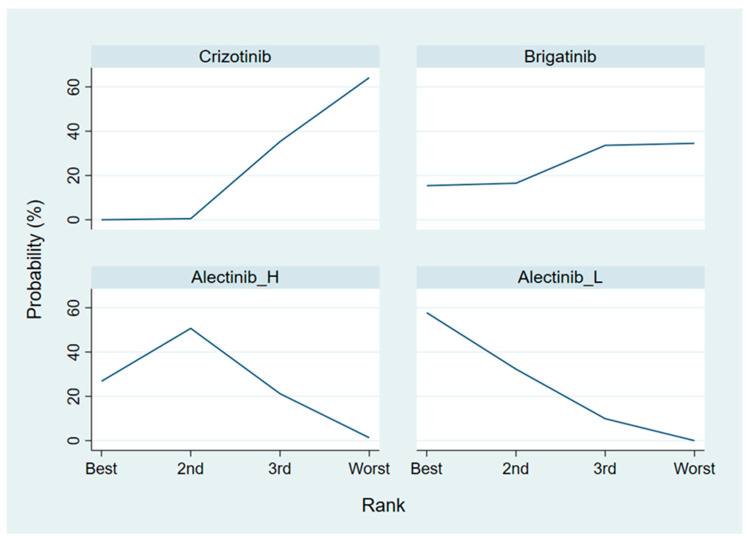
Probability of being the best treatment with regard to objective response rate (ORR).

## Appendix A

- The Embase database contains biomedical literature from 1974 to the present.
- MEDLINE and PubMed databases cover journals from 1966 to the present.
- Embase Classic: The Embase back file covers almost 2 million biomedical and pharmacological citations drawn from over 3000 international titles between 1947 and 1973.

**Table A1:** Search strategy in Embase.

Search	Query	Results
#1	‘non-small cell lung cancer’/exp OR ‘bronchial non-small cell cancer’ OR ‘bronchial non-small cell carcinoma’ OR ‘lung non-small cell cancer’ OR ‘lung non-small cell carcinoma’ OR ‘microcellular lung carcinoma’ OR ‘pulmonary non-small cell cancer’ OR ‘pulmonary non-small cell carcinoma’ OR ‘non-small cell bronchial cancer’ OR ‘non-small cell bronchial carcinoma’ OR ‘non-small cell cancer, lung’ OR ‘non-small cell lung cancer’ OR ‘non-small cell lung carcinoma’ OR ‘non-small cell pulmonary cancer’ OR ‘non-small cell pulmonary carcinoma’	171,940
#2	‘NSCLC’:ab,ti OR ‘nsclc’:ab,ti OR ((non NEAR/3 ‘small cell ‘ NEAR/3 lung NEAR/3 cancer):ab,ti) OR ((non NEAR/3 ‘small cell’ NEAR/3 lung NEAR/3 carcinoma):ab,ti) OR ((non NEAR/3 ‘small cell ‘ NEAR/3 lung NEAR/3 neoplasm):ab,ti)	115,898
#3	((‘ large cell ‘ NEAR/3 lung NEAR/3 cancer):ab,ti) OR ((‘ large cell ‘ NEAR/3 lung NEAR/3 carcinoma):ab,ti) OR ((‘ large cell ‘ NEAR/3 lung NEAR/3 neoplasm):ab,ti) OR ((squamous NEAR/3 lung NEAR/3 cancer):ab,ti) OR ((squamous NEAR/3 lung NEAR/3 carcinoma):ab,ti) OR ((squamous NEAR/3 lung NEAR/3 neoplasm):ab,ti) OR ((adenocarcinoma NEAR/3 lung NEAR/3 cancer):ab,ti) OR ((adenocarcinoma NEAR/3 lung NEAR/3 carcinoma):ab,ti) OR ((adenocarcinoma NEAR/3 lung NEAR/3 neoplasm):ab,ti)	11,061
#3	#1 OR #2 OR #3	182,051
#4	((‘anaplastic lymphoma kinase ‘ NEAR/3 inhibit*):ab,ti) OR ((‘anaplastic lymphoma kinase ‘ NEAR/3 antagonist*):ab,ti) OR ((‘ALK ‘ NEAR/3 inhibit*):ab,ti) OR ((‘ALK ‘ NEAR/3 antagonist*):ab,ti) OR ((‘alk ‘ NEAR/3 inhibit*):ab,ti) OR ((‘alk ‘ NEAR/3 antagonist*):ab,ti) OR ((‘ALKI’):ab,ti) OR ((‘ALKIS’):ab,ti)	3841
#5	crizotinib:ab,ti OR ‘pf 02341066’:ab,ti OR ‘pf 1066’:ab,ti OR ‘pf 2341066’:ab,ti OR pf02341066:ab,ti OR pf1066:ab,ti OR pf2341066:ab,ti OR xalkori:ab,ti OR ceritinib:ab,ti OR ‘ldk 378’:ab,ti OR ldk378:ab,ti OR ‘nvp ldk 378’:ab,ti OR ‘nvp ldk 378 nx’:ab,ti OR ‘nvp ldk378’:ab,ti OR ‘nvp ldk378 nx’:ab,ti OR zykadia:ab,ti OR alectinib:ab,ti OR ‘af 802’:ab,ti OR af802:ab,ti OR alecensa:ab,ti OR ‘alectinib hydrochloride’:ab,ti OR ‘ch 5424802’:ab,ti OR ch5424802:ab,ti OR ‘rg 7853’:ab,ti OR rg7853:ab,ti OR ‘ro 5424802’:ab,ti OR ro5424802:ab,ti OR brigatinib:ab,ti OR ‘ap 26113’:ab,ti OR ap26113:ab,ti OR lorlaninib:ab,ti OR ‘pf 06463922’:ab,ti OR lorlatinib:ab,ti OR ensartinib:ab,ti OR ‘unii sma5zs5b22’:ab,ti OR ‘370651 20 9’:ab,ti OR entrectinib:ab,ti OR ‘nms e 628’:ab,ti OR ‘nms e628’:ab,ti OR ‘rxdx 101’:ab,ti OR rxdx101:ab,ti	5932
#6	#4 OR #5	7335
#7	#3 AND #6	4922
#8	‘clinical trial’/de OR ‘randomized controlled trial’/de OR ‘randomization’/de OR ‘single blind procedure’/de OR ‘double blind procedure’/de OR ‘crossover procedure’/de OR (‘randomi?ed controlled’ NEXT/1 trial*) OR rct OR ‘randomly allocated’ OR ‘allocated randomly’ OR ‘random allocation’ OR (allocated NEAR/2 random) OR (single NEXT/1 blind*) OR (double NEXT/1 blind*) OR ((treble OR triple) NEAR/1 blind*)	1,760,209
#9	#7 AND #8	793
#10	#9 NOT ([adolescent]/lim OR [child]/lim OR [preschool]/lim OR [school]/lim)	786

* is truncation function in Embase.

**Table A2:** Search strategy in the Cochrane Library (CENTRAL and CDSR).

Search	Query	Results
#1	(‘bronchial non-small cell cancer’ OR ‘bronchial non-small cell carcinoma’ OR ‘lung non-small cell cancer’ OR ‘lung non-small cell carcinoma’ OR ‘microcellular lung carcinoma’ OR ‘pulmonary non-small cell cancer’ OR ‘pulmonary non-small cell carcinoma’ OR ‘non-small cell bronchial cancer’ OR ‘non-small cell bronchial carcinoma’ OR ‘non-small cell cancer, lung’ OR ‘non-small cell lung cancer’ OR ‘non-small cell lung carcinoma’ OR ‘non-small cell pulmonary cancer’ OR ‘non-small cell pulmonary carcinoma’):ti,ab,kw	13,321
#2	(‘NSCLC’ OR ‘nsclc’ OR ‘large cell lung cancer OR ‘large cell lung carcinoma’ OR ‘large cell lung neoplasm’ OR ‘squamous lung cancer’ OR ‘squamous lung carcinoma’ OR ‘squamous lung neoplasm’ OR ‘adenocarcinoma lung cancer’ OR ‘adenocarcinoma lung carcinoma’ OR ‘adenocarcinoma lung neoplasm’):ti,ab,kw	11,250
#3	#1 OR #2	15,086
#4	(‘anaplastic lymphoma kinase’ inhibitor OR ‘anaplastic lymphoma kinase’ antagonist OR ‘ALK inhibitor’ OR ‘ALK antagonist ‘ OR ‘alk inhibitor ‘ OR ‘alk antagonist ‘ OR ‘ALKI’ OR ‘ALKIS’):ti,ab,kw	429
#5	(crizotinib OR ‘pf 02341066′ OR ‘pf 1066′ OR ‘pf 2341066′ OR pf02341066 OR pf1066 OR pf2341066 OR xalkori OR ceritinib OR ‘ldk 378′ OR ldk378 OR ‘nvp ldk 378′ OR ‘nvp ldk 378 nx’ OR ‘nvp ldk378′ OR ‘nvp ldk378 nx’ OR zykadia OR alectinib OR ‘af 802′ OR af802 OR alecensa OR ‘alectinib hydrochloride’ OR ‘ch 5424802′ OR ch5424802 OR ‘rg 7853′ OR rg7853 OR ‘ro 5424802′ OR ro5424802 OR brigatinib OR ‘ap 26113′ OR ap26113 OR lorlaninib OR ‘pf 06463922′ OR lorlatinib OR ensartinib OR ‘unii sma5zs5b22′ OR ‘370651 20 9′ OR entrectinib OR ‘nms e 628′ OR ‘nms e628′ OR ‘rxdx 101′ OR rxdx101):ti,ab,kw	427
#6	#4 AND #5	646
#6	#3 AND #6	521 Source PubMed = 84 Embase = 356 clinicaltrials.gov = 45 ICTRP = 87
